# The Development, Implementation, and Feasibility of a Circadian, Light, and Sleep Skills Program for Shipboard Military Personnel (CLASS-SM)

**DOI:** 10.3390/ijerph19053093

**Published:** 2022-03-06

**Authors:** Elizabeth M. Harrison, Emily A. Schmied, Suzanne L. Hurtado, Alexandra P. Easterling, Gena L. Glickman

**Affiliations:** 1 Center for Circadian Biology, University of California San Diego, La Jolla, CA 92093, USA; a.easterling@alumni.albany.edu (A.P.E.); gena.glickman@usuhs.edu (G.L.G.); 2 Health and Behavioral Sciences Department, Naval Health Research Center, San Diego, CA 92106, USA; suzanne.l.hurtado.civ@mail.mil; 3 Leidos, Inc., San Diego, CA 92121, USA; 4 School of Public Health, San Diego State University, San Diego, CA 92182, USA; eschmied@sdsu.edu; 5 Institute for Behavioral and Community Health, San Diego, CA 92123, USA; 6 Departments of Psychiatry and Neuroscience, Uniformed Services University of the Health Sciences, Bethesda, MD 20814, USA

**Keywords:** circadian, sleep, light, education, outreach, military, operational

## Abstract

Service members face unique barriers to sufficient and high quality sleep. In the present study, a circadian, light, and sleep skills program for shipboard military personnel (CLASS-SM) was designed to encourage and inform strategies that support optimal sleep and circadian health in the context of those barriers. Phase 1 included program development and refinement via an iterative formative evaluation, including structured interviews with service members and feedback from veterans and experts, resulting in further tailoring to the population. In Phase 2, the highly tailored program was administered to shipboard personnel (*n* = 55), and acceptability indicators were measured. Sleep- and circadian-related knowledge (pre- and post-program) and the perceived relevance of, and satisfaction with, program content (post-program) were assessed. Before the intervention, most individuals were unaware that 7–9 h of sleep is recommended (72%) and had little understanding of the physiological effects of light; however, knowledge scores increased significantly post-program, from 51% to 88% correct (*p* < 0.0001). Reception was positive, with high reported satisfaction and relevance. Most individuals reported that they learned something new (89%), planned to use one or more learned strategies (100%), and intended to share learned information with others (85%); the physiological effects of light and circadian rhythms were the content areas most frequently reported as new and useful. The results demonstrate the need for, and feasibility of, the delivery of this program in operational environments.

## 1. Introduction

Sleep disruption is pervasive in the military. Recent studies of service members in operational environments indicate that over 50% of deployed service members suffer from chronic sleep restriction [1,2,3,4]. Sleep disruption in service members, however, is not limited to deployed settings. Non-deployed service members also face unique barriers to sleep, including non-standard and unpredictable schedules, hypervigilance, cultural views of sleep as a weakness, and habitual early-rise times [5,6,7,8,9,10,11,12,13]. Reviews synthesizing data across numerous military studies put average sleep durations for service members well below expert recommendations [8,13,14,15,16], and data from the 2018 Health Related Behaviors Survey of all non-deployed active duty service members indicates that, across all services, between 54–69% of individuals receive fewer than 6 h of sleep per night during the work week [17].

This lack of sufficient sleep has a marked negative impact on performance and health. Specifically, sleep restriction has been shown to impair service member performance on marksmanship and fitness tasks [18,19,20], and in two large-scale studies of service members in operational environments, insufficient sleep was associated with self-reported accidents or errors that affected the mission [1,2]. In addition to these acute, performance-related deficits, it is well established that restricted sleep has myriad long-term negative consequences on the physiological and psychological health of service members [21]. Demonstrated physiological issues associated with poor and/or insufficient sleep in service members include poorer self-rated general health [3,8], more healthcare utilization and lost work days [3,22], increases in body mass index and waist circumference [19], increases in inflammatory biomarkers [23], and increased rates of infection and diabetes [19,24]. Further, accumulating evidence suggests that sleep problems often precede and increase the risk of developing psychological disorders, including PTSD and depression, in these military populations [8,10,22,23,25,26].

The wealth of research demonstrating the significant adverse effects of sleep disruption in the military indicates a critical need for tailored, pre- or non-clinical interventions to prevent or minimize these issues. Additionally, it has been argued that education is a critical component of any shiftwork countermeasure program [27]. In fact, studies suggest that educational programs focused on behaviors that support healthy sleep may be useful in and of themselves, as behaviors that are associated with sleep problems, such as the use of technological devices before bed, are highly prevalent [28], while increased sleep knowledge has been related to better sleep habits in service members [29,30]. Consistent with those findings, a 2015 RAND study of sleep in the military identified a need for education regarding the importance of sleep and healthy sleep behaviors [8]. Though very limited prior work has empirically evaluated educational, sleep-focused interventions in service members, a recent effort to educate leadership on sleep was well received [31], and sleep education intervention studies in clinical populations and other elite occupational groups show promise, with myriad demonstrated improvements, including increased knowledge [32] and sleep health [33,34,35], decreased fatigue [36], and reductions in injuries and sick days [37].

Importantly, current research suggests that service members are likely to be receptive to educational interventions that aim to support better sleep health. Three recent studies indicate high motivation to improve sleep in U.S. Naval personnel, in particular, with the vast majority (>90%) recognizing that sleep is important to health and performance [30,38] and choosing to utilize optional intervention components to help improve sleep [39]. Similarly, in a study from the U.S. Army, over 80% of a sample of soldiers with insomnia reported interest in learning about good sleep habits and/or behavioral skills to improve their sleep [40]. In this same study, 59% indicated they would either prefer to try behavioral treatment before medications, or to forego medications entirely in favor of behavioral treatment.

The need and support for sleep education programs for service members is clear [1,8,41,42]. Yet, relatively little is known about current sleep-related knowledge or sleep hygiene practices among service members; however, the subject is gaining attention [11,28,29]. Without a thorough understanding of what service members already know about sleep and best practices, or what strategies they already use to improve sleep, the ability to develop educational interventions of value is limited. Further, as service members are a notoriously hard-to-reach population, information on the practical considerations surrounding delivery of educational interventions is needed.

A recent umbrella review of sleep health promotion interventions revealed most health promotion programs do not adequately describe the intervention, including its development, nor do they include health behavior change theories in that development [43]. More concretely, most sleep health promotion interventions do not consider factors critical to influencing sleep health according to the principals of behavioral science, such as knowledge or receptivity [43]. To address these gaps, an in-person, interactive sleep- and circadian health-focused program (“CLASS-M”) was developed, not only for, but with, substantive guidance from active duty service members, and further refined for shipboard military personnel (“CLASS-SM”). The goal of this study was to develop and evaluate the acceptability and feasibility of a brief, educational program to improve sleep in U.S. Naval shipboard personnel. To that end, we describe the development of, delivery of, and response to, the program, as well as its immediate impact on sleep and circadian knowledge, as assessed in a pilot trial, according to the GUIDED consensus statement and checklist for reporting intervention development studies in health research [44], and the TIDieR framework for publishing detailed descriptions of health interventions [45]. Preliminary outcomes data were positive and have been presented elsewhere [46]; a more comprehensive report of those findings will follow in a separate publication, including the longer-term impacts of this program on sleep quality, sleep-promoting behaviors, and knowledge retention. The pilot results reported here serve to further elucidate the unique needs of service members and highlight specific considerations for implementing educational interventions in this at-risk group.

## 2. Materials and Methods

### 2.1. Study Design

The study included two phases. Phase 1 consisted of a formative evaluation of initial program content. In Phase 2, the program was delivered to the target population in order to assess feasibility and acceptability, and to determine its effect on the knowledge of sleep and circadian health. GUIDED reporting items for development studies, such as this one, include reporting on the context; purpose; target population; intervention development approach; how existing published theory informed the process; use of any components from an existing intervention; guiding principles; stakeholders; changes in content and format from the start of the process; any changes required or likely to be required for subgroups; uncertainties at the end; and use of the TIDieR framework, all in an open access format [44,45].

### 2.2. CLASS Program Description

The foundation for the sleep education program was derived from an original slide-based lecture, Circadian, Light, and Sleep Skills, or “CLASS”, developed by the authors (EMH and GLG) to address sleep, circadian rhythms, and the physiological effects of light for college students [47], many of whom also fail to obtain sufficient sleep [48]. Original content areas included general information on sleep and circadian rhythms; the importance of sleep for academic performance; and healthy sleep behaviors, with associated concrete tips.

The content and structure were informed by leading sleep therapies (e.g., CBT-I, see exceptions in Section 2.3 below); the instructional design framework for health promotion applications (e.g., tailoring the message to knowledge and values, demonstrating observable effectiveness, making desired behaviors attainable and easy to understand, and enhancing the retention and transfer of knowledge) [49]; and health behavior change theories (e.g., Health Belief Model ((HBM); [50]). HBM posits that individuals are more likely to engage in health-promoting behaviors if they believe the benefits of the behavior outweigh the risks associated with not engaging in the behavior, if they feel personally at risk for adverse outcomes associated with the behavior, and if they have confidence in their ability to change. Accordingly, the intervention was designed to include real-world examples to inform participants of the benefits of engaging in healthy sleep behaviors and the risks of inadequate sleep, while also increasing their confidence by teaching them practical strategies to improve sleep and circadian health. The content of the program is reinforced by encouraging the use of a variety of different evidence-based behavior change techniques, including goal-setting, self-monitoring, and recognizing and altering environmental barriers to sleep (e.g., light and noise).

In this study, the original CLASS program was adapted for military populations and renamed “CLASS-M” (CLASS-military version). The CLASS program was adapted in the following three ways, through an iterative process that involved receiving feedback from military personnel, described below: (1) the adaptation of existing content and materials, (2) the addition of reinforcing text messages, and (3) the modification of length. The resulting program consisted of a tailored presentation (including PowerPoint slides) and a series of text messages designed to reinforce the content over time.

### 2.3. Adaptation of CLASS Content Areas

The original program content was tailored in several ways to meet the needs and norms of service members, which have been reported in previous studies [8,11,51]. First, in order to obtain buy-in and increase cultural relevance of the program, quotes from former service members regarding the importance of sleep for performance were added throughout the presentation, as were military-relevant images and examples. For instance, one figure showed the relationship between sleep and accidents using data derived from military samples. Additionally, a focus on physical performance was incorporated. As high value is placed on physical fitness and maximizing performance within the military culture, it was hypothesized that explaining the relationship between sleep, circadian health, and physical performance may increase participant buy-in. While “sleep hygiene” recommendations typically include regular exercise for sleep health [52], CLASS-M focused particularly on optimal timing of exercise as well as its direct effects on the biological clock [53,54].

**Figure 1:**
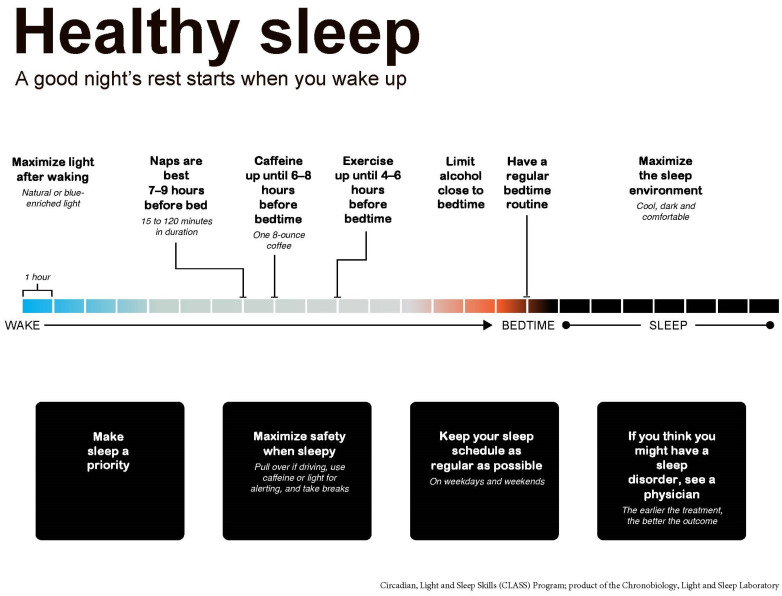
CLASS program overview. The novel circadian, light and sleep skills class was structured to provide knowledge, motivation, and concrete tips for improving sleep and circadian health across an entire waking day, beginning with the statement that “A good night’s rest starts when you wake up.” The novel circadian, light and sleep skills class for shipboard military personnel (CLASS-SM) described in this manuscript was subsequently developed to address the specific sleep, circadian, and alertness concerns of shipboard service members.

Second, CLASS-M incorporated information about how to adapt to non-standard sleep schedules and mitigate fatigue, which are critical issues in this shift-working population. Generally, the advice was designed to be brief, practical, and to address operational demands while still remaining as evidence-based as possible. To do this, the presentation began with an explanation of the underlying chronobiological processes that make adjusting to non-standard schedules difficult [55], then provided strategies participants could use to facilitate the adjustment, such as acute alerting and phase-shifting by light [56,57,58,59,60,61], strategies not often included in typical “sleep hygiene” recommendations [52]. This was built into the structure of the presentation, which was organized by time of day rather than only focusing on the time around sleep, to prompt participants to think about sleep in a different way. The program began with the maxim, “A good night’s sleep begins when you wake up,” and included the directive to obtain a potent light signal (bright and/or blue-enriched) upon waking; to use bright light exposure as a potential countermeasure for sleepiness throughout the day; and to minimize light (via intensity and spectrum) at bedtime (Figure 1). For ease of comprehension and application, “bright” was defined as brighter than typical indoor lighting, and examples of “blue-enriched” light included natural sunlight or any white light source with a relatively larger proportion of energy in the blue-wavelength region of the spectrum. In addition, the optimal timing of other activities that can impact sleep and circadian health, such as caffeine and alcohol consumption, napping, and exercise, were all described within the sequence of daily activities. Due to the extreme sleep restriction that is common in this population, in contrast to CBT-I and/or sleep hygiene recommendations, which include avoiding daytime napping, napping to compensate for severe decrements in sleep was encouraged; however, preferred timing and duration to minimize sleep disruption at bedtime were explained [62]. Importantly, the reference to specific clock times was avoided, and all behaviors were discussed in reference to desired sleep/wake times.

Third, tips for optimizing sleep quality, given sleep restriction in high operational-tempo settings, were provided. These included strategies for minimizing environmental factors that could impede sleep at bedtime (e.g., using white noise or earplugs to reduce noise). Fourth and finally, the CLASS-M program was designed to be interactive, with discussion prompts, quizzes, and a participatory introduction to progressive muscle relaxation.

### 2.4. Reinforcing Text Messages

Beginning one week after program delivery, four weekly text messages, designed to be brief and to reinforce concepts discussed in the program, were sent to each participant via the phone numbers provided to study staff at consent. The four below were selected out of a list developed by the authors due to their perceived importance and their relative independence from a conceptual standpoint.

- Week 1: Sleep is important to both physical and mental functioning. Make sleep a priority, as best as you can, wherever you can.
- Week 2: To help you feel more alert when you wake up, expose yourself to bright light right after waking, using sunlight or artificial blue-enriched light.
- Week 3: If needed, caffeine is best earlier in the day, and should be avoided (if possible) in the 6 h before bedtime, to prevent trouble falling asleep later.
- Week 4: Having trouble falling asleep? Try a muscle relaxation exercise, such as the one you learned in the sleep CLASS.

### 2.5. Program Length

The original CLASS and adapted CLASS-M programs were designed to be one hour in length. As noted below, the final in-person, expert-led component of the CLASS-SM program was 30 min, following a formative evaluation (with an intermediary 45 min version that briefly existed during Phase 1).

### 2.6. Participants and Recruitment

Phase 1 participants were recruited from local Navy Commands via fliers and in-person announcements. For Phase 2, command permission was obtained to board a docked Littoral Combat Ship (LCS) on two consecutive days for recruitment and program delivery. All participants provided written consent to participate, and all study procedures were approved by the Institutional Review Board of the Naval Health Research Center (NHRC.2017.0010).

### 2.7. Phase 1: Formative Evaluation of CLASS-M and Evolution to CLASS-SM

#### 2.7.1. Methods

The formative evaluation of CLASS-M consisted of an iterative revision process performed with service members and subject matter experts. First, the program was presented on three separate occasions to approximately 20 veterans, military dependents, and behavioral scientists. After each presentation, participants provided feedback on the content and format in informal group discussions. Feedback was incorporated into the program, and the presentation was repeated with a mixture of new and original audience members. Changes made as a result of the feedback received in these information presentations included: reducing the program time to 45 min, replacing certain scientific jargon with more Navy-relevant expressions and examples, increasing the repetition of key points to maximize knowledge retention, and using “sleep as a tool” to be a better sailor (and thus, an “S” was added for “shipboard,” resulting in CLASS-SM in Phase 2, see below). The refined program was then delivered in a one-on-one setting to five active duty service members, again following an iterative process with the program content tailored after each presentation.

#### 2.7.2. Measures

The participants completed a brief survey and a one-on-one interview immediately following the presentation. The survey asked participants to rate their satisfaction with the program length, relevance of the material, and program overall; in the interview, open-ended questions included, “What did you think about the presentation?” and “What suggestions do you have for how we could make it maximally relevant to Sailors?” Participants also reported demographic characteristics on the survey.

### 2.8. Phase 2—Feasibility and Acceptability of CLASS-SM

#### 2.8.1. Methods

The CLASS-SM program was presented to the crew of a Littoral Combat Ship (LCS) (*n* = 55 total; 6 females). The program was presented twice due to space limitations and to accommodate crew members’ varying schedules. Immediately prior to the program sessions, the participants completed the baseline questionnaire on computer tablets (T1). Then, presentation materials were loaded to a computer monitor provided by the ship, and the study team administered the program in a group setting. Immediately following the completion of the presentation, the crew completed a follow-up questionnaire (T2). Next, in each of the first four weeks following the presentation, a text message reinforcing a different content area was distributed to all study participants who provided contact information.

#### 2.8.2. Measures

*Personal Characteristics.* Basic demographic and service history data were collected, including age, education, sex, rank, and deployment history.

*Sleep-related knowledge* was assessed using seven items developed for this study, covering topics addressed in the program, such as “How many hours of sleep per night are recommended for adults?” and “If you are trying to adjust to a new timezone, when during the “new” day is it most important for you to get bright light?” Knowledge scores were generated as a percent correct, and the difference across timepoints was computed.

*Acceptability indicators (program relevance and satisfaction).* Post program (T2), participants reported the perceived relevance of the three major content areas of the program (general sleep, circadian rhythms, and sleep-promoting behaviors). The responses ranged from 1 (not at all relevant) to 7 (extremely relevant). Additionally, participants reported overall satisfaction with the program as well as its length, format, content, and coverage. The responses ranged from 1 (extremely dissatisfied) to 7 (extremely satisfied). The participants were also asked (free response) if they learned something new, if they were likely to use that information, and if they were likely to share that information. General feedback regarding program improvement was also solicited through a fourth free-response question: “Do you have suggestions for how this program could be improved?”

### 2.9. Analysis

All analyses were conducted in SPSS Statistical Software Version 24 (IBM, Corporation, Armonk, NY, USA) or GraphPad Prism (GraphPad Software, Inc., San Diego, CA, USA). Descriptive statistics were computed for the questionnaire data, and content analysis of the interview data was conducted for Phase 1. For Phase 2, repeated measures analyses were used to examine potential changes in sleep-related knowledge across time points (T1–2). Degree of satisfaction with and relevance of the program (T2) were determined with one-sample *t*-tests against neutral values (e.g., 4 on a scale of 1–7). These test the null hypothesis that scores in the sample are equal to the midpoint (median) of the test variable, in this case a neutral (neither positive nor negative) opinion on program satisfaction or relevance, equal to 4. All statistical tests were evaluated at the alpha = 0.05 level unless otherwise specified.

## 3. Results

### 3.1. Phase 1—Participant Characteristics and Feedback

Most (80%) of the five Phase 1 participants were Navy (versus 20% army), on active duty (80% versus 20% reservists), and male (80%). Suggestions received at this point included focusing even more on branch-specific experiences, such as shipboard life, as opposed to the military more generally; making the language less complex; the inclusion of more demonstrations and specific examples; focusing even more on the relationship between performance and sleep; and shortening the program further, to 30 min. As mentioned earlier, the final program was renamed CLASS-SM in light of the increased tailoring for shipboard service members.

### 3.2. Phase 2—Participant Characteristics

The majority of participants (86%) were between 25–39 years of age, and most (78%) ranked between E4–E9 (see Table 1 for more details).

### 3.3. Sleep-Related Knowledge

**Figure 2:**
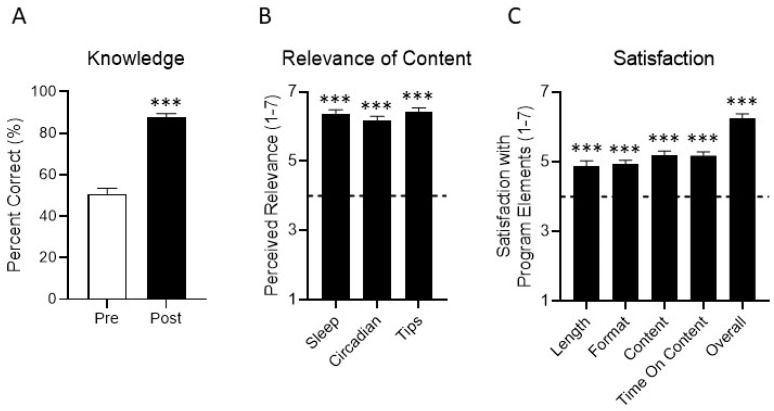
Knowledge, perceived relevance, and satisfaction. (**A**) Knowledge was assessed before and immediately after the program using seven items developed for this study, covering topics addressed in the CLASS-SM program. (**B**) Immediately following the presentation, participants reported the perceived relevance of the three major content areas of the program (general sleep, circadian rhythms, and sleep-promoting behaviors/tips). Responses ranged from 1 (not at all relevant) to 7 (extremely relevant). (**C**) Additionally, participants reported satisfaction with the program overall, as well as its length, format, content, and coverage. Responses ranged from 1 (extremely dissatisfied) to 7 (extremely satisfied). The horizontal dashed lines in panels (**B**,**C**) represent neutral values (a “4” on each scale). *** = *p* < 0.0001.

Knowledge increased from baseline to immediately following the program presentation (from 51% to 88% correct; t_(50)_ = 9.39, *p* < 0.0001; Figure 2A and Table 2). At T1, the most commonly missed items were the number of recommended hours of sleep, followed by the question on the potency of short wavelength (blue) light and how long it takes the circadian system to adjust to timezone or schedule changes. The other four questions were above 60% correct. Notably, a majority (72%) of participants believed a minimum of 6 or fewer hours to be included within the recommended number of hours of sleep for adults (mode response was 6–8 h (*n* = 24); see Table 2 for knowledge questions, answers, and percent correct for each item at T1 vs. T2 (*n* = 54)).

### 3.4. Acceptability Indicators (Program Relevance and Satisfaction)

Participants were significantly more satisfied than neutral on all aspects of the program (length, format, content, coverage; all means > 4 on a 7-point scale, all *p* < 0.0001; see Figure 2B), including high satisfaction with the program overall (*p* < 0.0001). Participants reported that each of the three major focus areas of the program (general sleep, circadian rhythms, and sleep-promoting behaviors) were highly relevant (all means > 4 on a 7-point scale, all *p* < 0.0001; Figure 2C).

**Figure 3:**
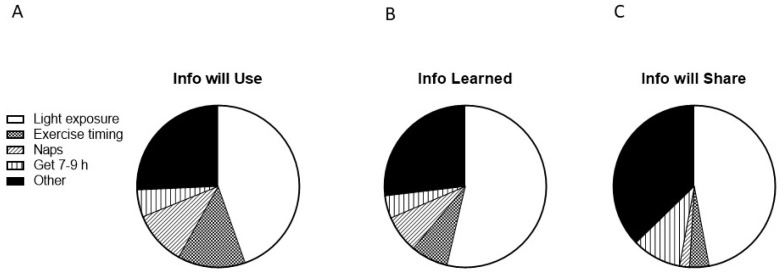
Program information individuals plan to use, learned, and plan to share with others. Individuals were asked (free response) (**A**) what information they planned to use going forward, (**B**) what information was new to them, and (**C**) what information they would share with others. Light exposure included any comment about modulating light exposure (for instance, intensity, wavelength, and duration), for the purposes of improving either sleep or alertness/performance; see Section 3.4 for examples. The “other” category comprises responses, which do not fit into the four most-frequently endorsed categories of learned material (light exposure, exercise timing, naps, and obtaining 7–9 h of sleep per 24 h), such as the timing of caffeine or alcohol consumption, or relaxation techniques.

When asked if they learned anything new in the program, 89% said yes, and 100% of those reported that they would use one or more newly learned strategies. The most frequently reported specific strategies for future use were optimizing light exposure, either for alertness or sleep (44.6%), exercise timing (13.5%), and naps (10.8%; Figure 3A). When prompted to list at least one new thing they learned, the most frequently reported content areas followed a similar pattern (Figure 3B). Some examples of free-response answers included “how light affects sleep,” “get an app to change the blue to more red light”, “dark during bed time”, and “I did not know how important the morning light is”.

When asked if they planned on sharing any of the information learned with anyone, 85% said yes. Of these, 33 said they would share with their spouse, 27 said other family members, 22 said friends, and 15 said coworkers. When asked what information they would share, information about light was the most frequent response (47.1%; Figure 3C).

Additionally, individuals were asked if they had any recommendations for improvement for the CLASS-SM program. Twelve individuals (53% of those who responded to the item) endorsed “none;” five of those individuals used the space to provide positive feedback (e.g., “all the information was great,” “awesome job,” “good information,” “information was very informative,” and “great job”). Eleven individuals had a recommendation for change. These included adding more citations/context for the presentation (*n* = 2), conducting the presentation off-ship (*n* = 2), speaking more to the military culture (*n* = 2), and providing more resources (*n* = 2). Additionally, one individual suggested receiving input from other commands, a second wanted more discussion about sleep deficits, and a third individual made a comment about disliking slide presentations as a format.

## 4. Discussion

Despite substantial evidence that service members experience high rates of sleep issues, few interventions have been developed to meet their unique needs. Following the guidance for reporting intervention development studies in health research (GUIDED) consensus study guidelines [44], this paper provides initial evidence of the feasibility of implementing a novel Navy-specific, sleep-focused educational program in a shipboard setting. The implementation of interventions for enhancing sleep and circadian health is under-studied and under-reported, but may profoundly affect uptake and even efficacy [71]. A recent umbrella review of sleep promotion interventions highlights the need for a focus on behavior change theory in the development of sleep health interventions, as well as for measures of feasibility and receptivity [43]. The research team successfully delivered the program within the confines of the ship’s operating environment and schedule. The sailors were highly receptive to the program itself, consistent with a recent study wherein service members were open to behavioral interventions for sleep issues [40].

In addition to providing evidence of feasibility, the results of this study show that even a very brief educational program, such as CLASS-SM, can address gaps in sleep-related knowledge among service members. Further, the results indicate particular content areas in which knowledge may be lacking in this population; the circadian content of the program was perceived as more novel and salient than the sleep content. For example, members appear to have limited prior knowledge of the importance and effects of light related to sleep but they view light manipulation as a feasible strategy to improve their sleep. Specifically, the importance of light and its timing was the most commonly reported new concept learned (54%), new strategy that would be used to improve sleep (45%), and concept to share with others (47%; Figure 2). In contrast, service members less frequently reported that the content related to the importance to other activities (e.g., exercise, consumption of caffeine and alcohol, naps, and the use of electronics) was novel, or that they intended to use strategies to address those behaviors (all under 13%). This indicates that the choice to focus on the effects of light on the circadian system and to structure the class across time of day successfully addressed a knowledge gap.

Light, noise, temperature, and air pollution were recently identified as primary environmental barriers to sleep in operational contexts [9,11,51]. A central theme of the CLASS-SM program is to acknowledge these and other barriers to sleep health in operational contexts, and to then instruct participants not to forego all healthy sleep practices, but rather do what they can, when they can. In other words, while certain behaviors (such as keeping a regular bedtime) may be difficult or even impossible in an operational environment, there are some factors that remain under individual control, such as the timing of caffeine intake in relation to bedtime [72].

Additionally, that a majority of the participants reported that they would share new information and strategies with their families, spouses, friends, and/or coworkers is promising and may serve to improve outcomes via indirect reinforcement of newly learned concepts. Delivering the CLASS-SM program to an entire unit or ship may also have a similar reinforcing effect.

The results of this study should be interpreted while taking into consideration several limitations. First, all participants were stationed aboard a single ship, which could limit the generalizability of the findings [73], considering that the schedule, environment, and social norms surrounding sleep could greatly vary across different types of ships or missions [74]. Additionally, this study focused on program development, acceptability and feasibility; thus, no control comparison or sleep outcomes data are reported here. While sleep and circadian researchers may be less familiar with the publication of intervention development, feasibility, and implementation results than public health or health promotion researchers, the value of these outcomes to the field should not be underestimated [43,71,75]. Intervention development studies have been recognized as the best means for improving study replicability and improving efficacy in applied research (e.g., [45,76]). Future efforts will include: expanding the sample size, a control comparison group, efficacy outcomes, a longer follow-up, and a more extensive deployment or underway period following exposure to the program. Second, this study utilized text-message reinforcements; however, these may not always be feasible, depending on location and environment. To our knowledge, no sleep health education program to date has augmented in-person interventions with text message reinforcements in military populations, though recent studies of telephone and text message sleep interventions demonstrated the capacity for these strategies to improve sleep quality and quantity in civilian populations [36,77,78]. Future work should include read receipts or response rates to assess delivery, dose, and efficacy. Additional considerations may include coordinating this program with another that educates leadership on their unique role in helping reinforce concepts and further promote healthy sleep practices in training and operational contexts [31,79]; early intervention and the involvement of leadership may be vital to combat some of the cultural barriers to sleep encountered in military settings [8,28,79]. Plans to examine data after a more prolonged follow-up period will determine if improvements are sustained.

## 5. Conclusions

These results indicate that the CLASS-SM program is both informative and well received—a promising result given the historical perception within the military that sleep is something service members can do without [8]. In the next step of this effort, a longer term follow-up is planned and will incorporate a more comprehensive examination of the effects of CLASS-SM on sleep, sleep-related behaviors, and psychological health.

## Figures and Tables

**Table 1:** Participant characteristics.

Characteristics	*n* (%)
**Gender**	
Male Female	49 (89.1) 6 (10.9)
**Age** 17–24 25–29 30–39 40+	3 (5.5) 15 (27.3) 32 (58.2) 5 (9.1)
**Race/ethnicity** White Black Hispanic Asian Other/multiple	26 (47.3) 9 (16.4) 10 (18.2) 3 (5.5) 7 (12.7)
**Marital status** Married/cohabitating Divorced/widowed Never married	31 (56.4) 7 (12.7) 17 (30.9)
**Paygrade/rank ^1^** E1–E3 E4–E6 E7–E9 Officer	2 (3.6) 25 (45.5) 18 (32.7) 10 (18.2)
**Education** High school Some college Two-year degree Four-year degree or higher	19 (34.5) 11 (20.0) 9 (16.4) 16 (29.1)

^1^ E = Enlisted. E1–E3 = apprenticeship, E4–E6 = petty officer, and E7–E9 = chief petty officer.

**Table 2:** Sleep and circadian knowledge questions.

Questions	Answers	T1 % Correct	T2 % Correct
The two primary regulators of sleep include the ____ and ____ systems.	**a. Circadian and homeostatic** [63].	61.1	96.3 ***
	b. Circadian and vestibular.		
	c. Homeostatic and vestibular.		
	d. Vestibular and visual.		
In order to optimize sleep and circadian health, short wavelength (blue) light:	**a. Is an important signal of day during** **active periods** [64].	20.4	79.6 ***
	b. Helps you to fall asleep when used close to bedtime.		
	c. Should be avoided at all costs.		
	d. Is meaningless. All wavelengths/colors of light affect our biology in the same way.		
Which of the following stages of sleep occurs more often in the second half of an eight-hour sleep episode?	a. NREM.		
	**b. REM** [65].	68.5	88.9 **
	c. Both, NREM and REM increase over the course of the sleep episode.		
	d. Neither, NREM and REM decrease over the course of the sleep episode.		
How many hours of sleep per night are recommended for adults? Between ____ and ____ hours	**7–9** [16,66]. (7–8 and 8–9 also accepted).	16.7	81.5 ***
If you are feeling sleepy, and then you go outside into the sunshine for half an hour, what might you expect to feel?	a. More sleepy.		
	**b. Less sleepy** [57].	70.4	98.2 ***
	c. No difference.		
Approximately how long does it take your circadian system to adjust when you change your light schedule by three hours (by flying from one U.S. coast to the other)?	a. It adjusts immediately.		
	b. One day.		
	**c. Three days** [64].	40.7	74.1 ***
	d. One week.		
	e. Three weeks.		
If you are trying to adjust to a new timezone, when during the “new” day is it most important for you to get bright light?	a. In the “new” morning * [67,68].	77.8	94.4 *
	b. In the “new” afternoon.		
	c. In the “new” evening.		
	d. In the “new” night.		

**The text in bold** indicates the correct answer(s); asterisks indicate paired *t*-test results (*n* = 54); * = *p* < 0.05, ** = *p* < 0.01, *** = *p* < 0.001. * While there is no current consensus on the best way to use light for the treatment of jet lag [69], and light during local morning may be theoretically contra-indicated under certain conditions, this was the advice provided based on existing evidence and on a need to maintain alertness under operational schedules [70].

## Data Availability

The data that support the findings of this study are available from the authors but restrictions apply to the availability of these data, which are not publicly available. Data are however available from the authors upon reasonable request and with permission of the U.S. Government.

## Disclaimer

Suzanne Hurtado and Gena Glickman are military service members or employees of the U.S. Government. This work was prepared as part of their official duties. Title 17, U.S.C. §105 provides that copyright protection under this title is not available for any work of the U.S. Government. Title 17, U.S.C. §101 defines a U.S. Government work as work prepared by a military service member or employee of the U.S. Government as part of that person’s official duties. Report No. 21–69 was supported by the Department of Defense Congressionally Directed Medical Research Programs, the Joint Program Committee-5 Early Assessment and Intervention Working Group, under work unit no. N1634, and the Fatigue Countermeasures Working Group, under award #66619. The views expressed in this article are those of the authors and do not necessarily reflect the official policy or position of the Department of the Navy, Department of Defense, nor the U.S. Government. The study protocol was approved by the Naval Health Research Center Institutional Review Board in compliance with all applicable federal regulations governing the protection of human subjects. Research data were derived from an approved Naval Health Research Center Institutional Review Board protocol, number NHRC.2017.0010.
